# Direct analysis in real‐time mass spectrometry: Observations of helium, nitrogen and argon as ionisation gas for the detection of small molecules using a single quadrupole instrument

**DOI:** 10.1002/rcm.9521

**Published:** 2023-05-03

**Authors:** Simone Mathias, Patrick Sears

**Affiliations:** ^1^ School of Chemistry and Chemical Engineering University of Surrey Guildford UK

## Abstract

**Rationale:**

Direct analysis in real time is typically performed using helium as the ionisation gas for the detection of analytes by mass spectrometry (MS). Nitrogen and argon are found with abundance in the air and provide a cheaper and greener alternative to the use of helium as ionisation gas. This study explores the use of helium, nitrogen and argon as ionisation gas for the detection of organic compounds.

**Methods:**

Four illicit drugs, two amino acids and five explosives were chosen as target analytes to understand selectivity, sensitivity and linearity when helium, nitrogen or argon was used as the ionisation gas with the direct analysis in real time (DART) source. Analysis was carried out on a Waters Acquity QDa single quadrupole mass spectrometer.

**Results:**

Calibration curves over the range of 5–100 ng were produced for each analyte using the different ionisation gases to assess the instrument response. Nitrogen gave a higher response to concentration than helium or argon; however, the lowest limits of detection were observed when helium was used.

**Conclusions:**

All the target analytes were detected using DART‐MS with helium, nitrogen or argon as the ionisation gas. Whereas helium provided the highest sensitivity, nitrogen produced reasonable limits of detection and had good linearity across the concentration range explored, suggesting it provides a greener and cheaper alternative to helium.

## 1 INTRODUCTION

Direct analysis in real time mass spectrometry (DART‐MS) was first introduced in 2005 by Cody et al.^1^ The original DART ion source uses a moderate pressure of gas (typically 500–700 kPa of helium or nitrogen) that flows through a chamber where an electrical discharge (created by applying high electrical potential to a needle) produces ions, electrons and excited state species within a plasma. The gas then passes through a heated region (50–500°C) before passing through a grid electrode, which can be biased to remove charged species (anions/cations and electrons). The heated exiting gas, containing electronically excited, neutral species, can interact with a sample in liquid, solid or vapour phase form before reaching the mass spectrometer inlet.^2^

Helium (He) is the most widely reported gas used with DART as its excited electronic state (2^3^S) has an energy of 19.8 eV, meaning it can readily react with atmospheric water (which has an ionisation energy of 12.62 eV).^3^ This type of ionisation, known as Penning ionisation, occurs due to an interaction between an electronically excited gas phase molecule (He*) and a target molecule (M). The collision results in the ionisation of the molecule, producing a cation M^+•^, an electron and a neutral gas molecule.

$${\mathrm{He}}^{*}+M\to M^{+•}+e^{-}+\mathrm{He}$$

Most positive ionisation for DART relies on helium reacting with atmospheric water, which in turn undergoes further ion‐molecule reactions to form protonated water clusters.^4^, ^5^

$${\mathrm{He}}^{*}+H_{2}O\to H_{2}O^{+•}+e^{-}+\mathrm{He}$$

$$H_{2}O^{+•}+H_{2}O\to H_{3}O^{+}+{\mathrm{OH}}^{-}$$

$$H_{3}O^{+}+{\mathrm{nH}}_{2}O\to {\left[{\mathrm{nH}}_{2}O+H\right]}^{+}$$

These water clusters can then act as secondary ionising species and generate ions through protonation, deprotonation, charge transfer and adduct formation. Protonation will occur only if the analyte has a higher proton affinity than the water cluster.^4^

$${\left[{\mathrm{nH}}_{2}O+H\right]}^{+}+M\to {\left[M+H\right]}^{+}+{\mathrm{nH}}_{2}O$$

Adduct formation can also occur with the use of additional reagents that can act as dopants, for example, ammonium hydroxide for the production of ammonium ions.^2^, ^5^

$${\left[NH_{4}\right]}^{+}+M\to {\left[M+NH_{4}\right]}^{+}$$

Negative ionisation is not well reported within the literature; however, several ionisation mechanisms have been proposed.^1^, ^6^, ^7^ The background mass spectrum for negative ion DART shows an abundance of O_2_^−•^, and it has been suggested that this occurs due to electrons generated by Penning ionisation being captured by atmospheric oxygen to form O_2_^−•^. This species can then react with an analyte by proton abstraction, charge exchange or attachment.^6^ For the following equations, G is a gas molecule.

$$\text{Proton abstraction}:{O_{2}}^{-•}+M\to {\left[M-H\right]}^{-}+{\mathrm{OOH}}^{•}$$

$$\text{Charge exchange}:{O_{2}}^{-•}+M\to M^{-•}+O_{2}$$

$$\text{Attachment}:{O_{2}}^{-•}+M\to {\left[M+O_{2}\right]}^{-•}*+G\to {\left[M+O_{2}\right]}^{-•}+G^{*}$$

Although helium is clearly advantageous due to its ability to readily ionise water molecules, it is expensive and not environmentally sustainable especially when compared to nitrogen (N_2_) and argon (Ar) which are found in abundance in the air. There are a growing number of publications on the use of nitrogen DART, as compounds with ionisation energies equal to or lower than 10.2 eV can be ionised effectively with nitrogen^8^ (which has been reported to have ionisation energies from 8.5 to 11.5 eV, with the possibility of some excited states reaching 15 eV).^9^, ^10^ Applications using nitrogen DART have included the analysis of designer drugs,^11^ pharmaceuticals^12^ and water contaminants in the international space station.^13^ The main reasons for choosing nitrogen over helium have been the cost and availability. A recent paper using DART with ion mobility spectrometry implemented nitrogen throughout the system which, in addition to reducing cost, also enabled a higher electric field to be applied, maximising the resolution of the mobility spectrometer.^14^ Heat transfer when using nitrogen is reduced (compared to helium)^13^; however, mixing with analytes in air is improved.^15^

Less literature is available surrounding the use of argon as its ionisation energies for ^3^P_2_ and ^3^P_0_ are 11.55 and 11.72 eV, respectively, and therefore unable to ionise water which results in poor sensitivity without the use of dopants within the gas stream.^16^, ^17^, ^18^ The lower ionisation energy of argon with carefully chosen dopants has been exploited to selectively ionise analytes in the presence of an interferent.^19^

DART has many published applications in fields including forensics, pharmaceuticals, environmental, food and beverages and cosmetics.^20^, ^21^, ^22^, ^23^, ^24^, ^25^ Ambient ionisation techniques allow for high throughput and rapid analysis of samples directly with minimal sample preparation, making them ideal for screening applications.^26^ For this reason, illicit drugs, amino acids and explosives were chosen as the target analytes. Amino acids were included in this study as they are widely used as health indicators, and the inclusion of some simple metabolomics‐type analytes demonstrates their potential capability of screening for this application. Amino acids are also zwitterionic and were included in this (and a wider study yet to be published) to explore ionisation mechanisms in both positive and negative modes. In addition, both ionisation modes can be explored with these analytes, as the drugs and amino acids ionise in positive ion mode, whereas the explosives tend to ionise in negative mode. The analytes have also been chosen to represent different ionisation routes (e.g., TNT can undergo proton abstraction or direct charge transfer to produce [M‐H]^−^ or [M]^•−^); they also have a range of thermal and electronic stabilities.^27^

## 2 EXPERIMENTAL

### 2.1 Materials

Optima LC–MS‐grade methanol, water and analytical reagent‐grade 96% ethanol were purchased from Fisher Scientific (Loughborough, UK). Leucine (99.89% purity) and phenylalanine (99.88% purity) were obtained from LGC (Teddington, UK). Amphetamine, ketamine, (−)‐trans‐Δ^9^‐tetrahydrocannabinol (THC) and cocaine were purchased from Merck (Ceriliant brand, Gillingham, UK). PETN, RDX, TNT, tetryl and HMTD were purchased from AccuStandard (Newhaven, CT, USA). Helium (99.996% purity), argon and nitrogen (99.998% purity) were obtained from BOC (Guildford, UK).

### 2.2 Sample and standard preparation

Phenylalanine and leucine were dissolved in water before dilution in methanol. All other standards were prepared volumetrically and diluted in methanol. Standards were prepared at the following concentrations 0.25, 0.5, 0.75, 1, 1.5, 2, 2.5, 5, 7.5, 10, 15, 20, 25, 50, 75, 100 μg/ml. These solutions were used to determine the limits of detection and to test the linear dynamic range (LDR) for each gas/analyte.

### 2.3 Experimental set‐up

**FIGURE 1:**
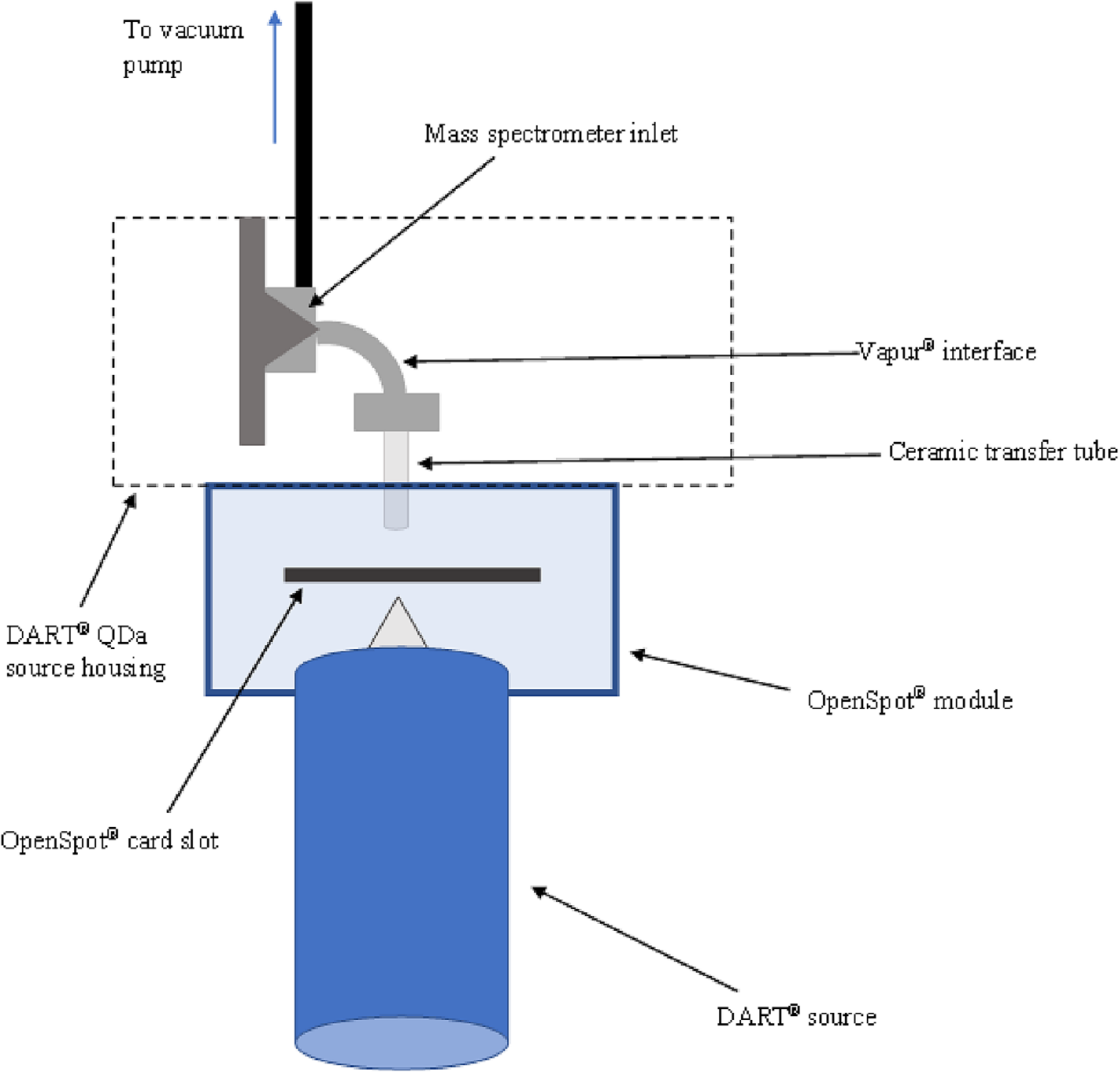
Schematic showing the direct analysis in real time (DART) source coupled to the Waters QDa mass spectrometer with the VAPUR interface and OpenSpot module [Color figure can be viewed at wileyonlinelibrary.com]

Analysis was performed using the Waters QDa mass spectrometer (Waters, Wilmslow, UK) with a direct analysis in real‐time source (IonSense, Saugus, MA, USA). An OpenSpot® module was fitted to the DART QDa source housing along with the Vapur® interface, and OpenSpot® samples cards (all IonSense (Saugus, MA, USA)) were used for sample introduction. One microlitre of the target analyte was deposited onto the wire mesh and the solvent left to evaporate leaving solid analyte residue. The system was operated in performance mode, with an external rotary pump (Vacuubrand RE 6) and 0.2 mm MS inlet aperture. The separate pump for the Vapur® interface was calibrated for optimum performance (1.3 turns) as per the Waters QDa Vapur® Interface User Manual. The front of the DART source was positioned at 1.4 cm on the source housing. Figure 1 depicts a schematic of the set‐up, and photographs can also be found in the supplementary information (Figures S1 and S2).

The source temperature was set to 150°C for all analyses, with the MS operating in positive mode for the following analytes: amphetamine, ketamine, THC, cocaine, phenylalanine, leucine and HMTD and in negative mode for the analysis of TNT, tetryl, PETN and RDX. Initial experiments were run in full scan over the mass range *m/z* 30–500, with a scan time of 0.15 s, and cone voltage was varied from 5 to 80 V for positive mode analytes (except HMTD) and 5–30 V for positive and negative ionising explosives to develop single ion monitoring (SIM) methods—details of which can be found in Table S1 (supporting information) along with the ions identified and their proposed elemental species.

The DART was operated with helium, nitrogen or argon as the ionisation gas, with nitrogen used when the DART was in “standby” mode. The grid electrode voltages were set to ±350 V. The temperature of the gas was optimised for positive and negative mode analytes and varied depending on the ionisation gas based on the largest signal intensity observed for the most abundant ion. Although the number of ions generated was temperature dependent, no other changes were observed in the ion signal indicating a lack of bias. Table 1 shows the optimised temperature setting for each analyte with the different ionisation gases.

**TABLE 1:** Analytes and the optimised ionisation gas temperature

Analyte	Gas temperature (°C)		
	Helium	Nitrogen	Argon
Amphetamine	200	250	200
Ketamine, THC, cocaine, phenylalanine	200	250	250
Leucine	200	250	200
HMTD	150	150	150
TNT, tetryl, PETN, RDX	200	200	200

## 3 RESULTS AND DISCUSSION

Initial argon experiments conducted without the presence of ethanol as a dopant resulted in no background or analyte ions formed over the mass range *m/*z 30–500, supporting the observations of Cody and Dane.^17^ Where dopants have been used by other researchers, in both cases, a syringe pump was used to introduce the dopant solvent to the gas stream produced by the DART.^17^, ^18^ This experimental set‐up was impractical due to the constraints of the OpenSpot® enclosure. Instead, a small reservoir of ethanol was placed in the semi‐enclosed region to allow a headspace of ethanol vapour to form. This enabled the argon to ionise ethanol molecules within the atmosphere and produce a background spectrum. Figures S3 and S4 (supporting information) show the background spectra for all gases. Compared with the other gases, argon produced the lowest background signal with helium producing the highest. This observation is to be expected, due to the higher energy of the excited electronic state of helium.

**FIGURE 2:**
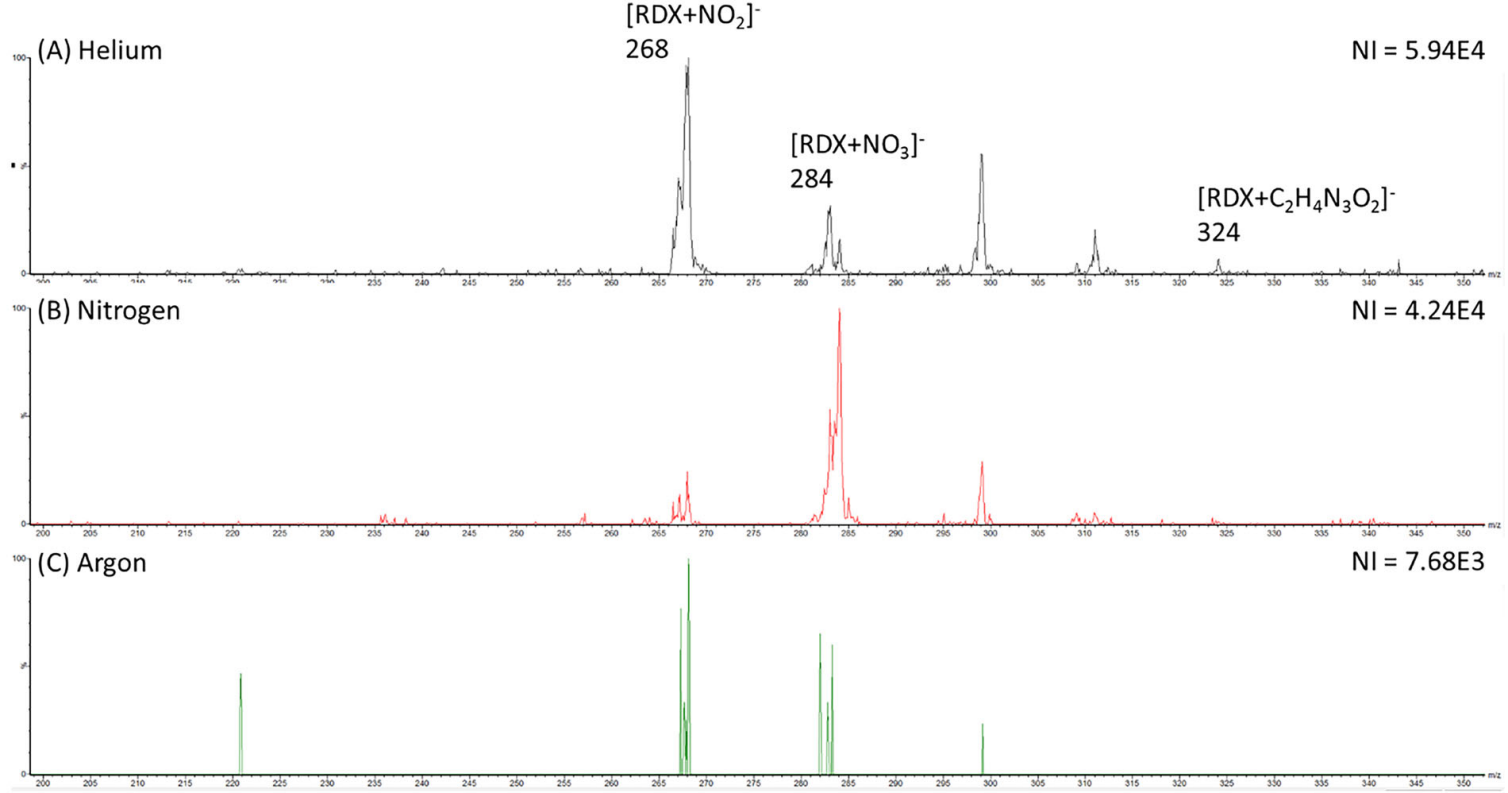
Mass spectrum for 100 ng of RDX produced with A, helium, B, nitrogen and C, argon as the direct analysis in real time (DART) ionisation gas with the ions relating to RDX identified and the number of ions (NI) for most abundant peak [Color figure can be viewed at wileyonlinelibrary.com]

It has been previously reported by Yang et al that DART used with argon gas as opposed to helium can result in minimised fragmentation within the spectra, overcoming the difficulties with the identification of the molecular ion from the fragment ions.^18^ When comparing spectra of the explosive analytes, those produced by argon and helium were similar in terms of the ions produced as seen in Figure 2; with nitrogen, however, there was a difference in which ion was detected as the most abundant. The same observation was made by An et al when using helium, nitrogen and argon for the detection of explosives.^16^ Figure 2 shows an example of mass spectra for RDX where the ion *m/z* 268 identified as [RDX + NO_2_]^−^ is the most abundant ion for both helium and argon; however, the ion *m/z* 284 attributed to [RDX + NO_3_]^−^ dominates the spectra for nitrogen DART. This suggests that the ionisation process for helium and argon in negative mode is similar, whereas it has been proposed that negative ionisation with nitrogen has a highly oxidative environment.^16^ In addition, within the helium and nitrogen spectra, an ion at *m/z* 324 can be identified as [RDX + C_2_H_4_N_3_O_2_]^−^, which occurs as a result of an intact molecule of RDX breaking down into a fragment [C_2_H_4_N_3_O_2_]^−^ seen at *m/z* 102 (not pictured), which in turn forms an adduct with intact RDX. With argon, it is possible that the softer ionisation prevents the formation of this fragment, meaning the adduct at *m/z* 324 is absent from the spectra; however, it is also likely that the signal at *m/z* 324 is very weak and therefore not observed (below the instrumental detection limit). A corresponding example of positive mode spectra can be seen in Figure S5.

Ambient ionisation MS techniques are often most suited for qualitative research or data collection due to their minimal sample preparation and rapid data outputs. These features are attractive for screening applications where a semi‐quantitative result providing an estimated concentration is beneficial.^28^, ^29^, ^30^, ^31^ To gain a better understanding of the performance and subsequent differences of each ionisation gas, both linearity and limits of detection (LOD) were explored as they offer a simple means of comparison.

When processing the data, the most abundant ion for each analyte was used for linearity plots and for calculating LODs to show a best‐case scenario for each technique. Table S1 (supporting information) reflects which ion was the most abundant for the target analytes. Our previous research using atmospheric solids analysis probe and secondary electrospray ionisation for the detection of explosives showed that changing ion source can result in different ions being observed, and therefore, using the most abundant ion was deemed the most appropriate method of comparison.^32^

**FIGURE 3:**
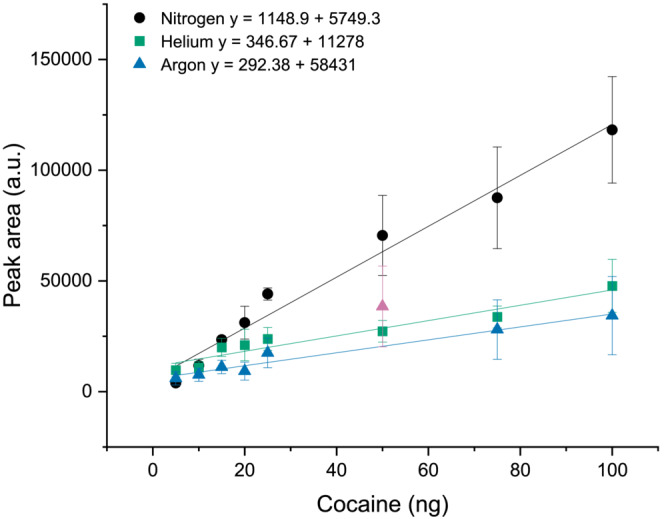
Calibration curves (n = 5) for cocaine using nitrogen (black circles), helium (green squares) and argon (blue triangles with pink triangle noted as an outlier) as the ionisation gas with direct analysis in real time (DART). Error bars are equal to ±1 standard deviation [Color figure can be viewed at wileyonlinelibrary.com]

Figure 3 shows the LDR established for cocaine using helium, nitrogen and argon as the ionisation gas. The remaining LDR for each analyte are presented in Table 2. The maximum concentration tested was 100 ng, and the full LDR may extend beyond the data presented here.

**TABLE 2:** Linear dynamic ranges (LDR) and correlation coefficients (*R* ^2^) for each analyte using direct analysis in real time (DART) with helium, nitrogen or argon as the ionisation gas

Analyte	Helium		Nitrogen		Argon	
	LDR (ng)	*R* ^2^	LDR (ng)	*R* ^2^	LDR (ng)	*R* ^2^
Amphetamine	5–100	0.9209	5–100	0.9480	5–100^a^	0.9565
Cocaine	5–100	0.9270	5–100	0.9762	5–100^a^	0.9593
Ketamine	5–100	0.9553	5–100	0.9149	5–100^a^	0.8990
THC	5–100	0.9587	5–75	0.9958	5–100^a^	0.9511
Phenylalanine	5–100	0.9852	10–100	0.9946	5–100	0.9830
Leucine	5–100	0.9753	10–100	0.9438	5–100	0.9636
HMTD	5–100	0.9687	5–100	0.9973	10–75	0.7518
PETN	10–100	0.9218	5–100	0.9875	20–100	0.9515
TNT	5–100	0.9766	5–75	0.9749	5–100^a^	0.8641
Tetryl	5–75	0.9141	5–100	0.9368	5–25	0.9375
RDX	5–100	0.9711	5–100	0.9961	5–100^a^	0.9819

^a^ A point was removed from the LDR.

A statistical test (detailed in the supporting information) was applied to the calibrations curves, and any points that were ±2 standard deviations from the line of best fit were deemed to be outliers and excluded from the LDR. Most outliers were observed when using argon; this may have occurred due to the inherent variability of DART amplified by a low sample response; however, outliers could also have occurred (for argon only) due to the need to refresh the reservoir of ethanol during the course of the analysis. Examples of calibration curves where outliers have been identified can be found in Figures S6–S11.

A general trend in terms of “good” linearity is observed for both helium and nitrogen gas, with argon performing the poorest, as shown by the *R* ^2^ values particularly for ketamine, HMTD and TNT, which are below 0.9000. It is also apparent that nitrogen tends to produce a higher response to concentration, as demonstrated by the slope of the line in Figure 3. This is surprising as it would be expected that nitrogen, having a lower energy excited state than helium, should produce fewer ionised species. This observation was also noted by Sisco et al who reported that nitrogen produced a signal two to three orders of magnitude higher than that of helium.^33^

The repeatability of these data is typical of an ambient ionisation technique using peak response, as shown by the error bars on the calibration curves, due to the manual nature of sample introduction.^34^ An internal standard could be used to improve the variability resulting of analyte placement on the OpenSpot® sample cards, efficiency of desorption from the surface of the sample card and repeatability of analyte deposition on the surface. The relative standard deviations (RSDs) calculated provide further support for the variability observed using peak area alone to process data. Table S2 (supporting information) shows the calculated RSDs for all analytes across all ionisation gases. For helium gas, RSDs can be observed from as low as 5%, with a maximum of 108%, but typically sit around ~30%–50%. A slight improvement is seen with nitrogen gas for most of the analytes with typical RSDs of ~15%–20%, with a minimum RSD of 3% and a maximum of 96%. Typical argon RSDs are observed ~40%–60% with the lowest RSD observed at 9% and the largest at 105%. These findings suggest that analysis performed with argon gas may be susceptible to data that are less repeatable compared with using helium or nitrogen as the ionisation gas. The use of a reservoir for introducing the dopant into the argon gas steam is likely an inefficient method, resulting in the poor reproducibility observed in this study and potentially some of the outliers observed.

The accuracy of DART is as expected of an ambient ionisation technique with no internal standard present. At lower concentrations, the detector response may be lower than predicted by the linear correlation. This can result in negative concentrations being calculated. Back‐calculated accuracy (%) for all analytes across all ionisation gases can be found in Table S3 (supporting information).

Estimated LODs can be seen in Table 3. Details of the calculation method for determining LOD can be found in the supporting information.

**TABLE 3:** Estimated limits of detection for analytes detected using direct analysis in real time (DART) with helium, nitrogen or argon as the ionisation gas

Analyte	Estimated limit of detection (ng)		
	Helium	Nitrogen	Argon
Amphetamine	0.1	2.5	0.3
Cocaine	0.07	0.5	0.4
Ketamine	0.03	0.2	0.0009
THC	0.3	1.5	0.5
Phenylalanine	3.0	10	4.0
Leucine	0.2	7.5	1.0
HMTD	1.1	1.8	7.5
PETN	0.5	0.04	1.5
TNT	0.5	2.0	2.0
Tetryl	0.1	0.5	0.1
RDX	0.01	0.03	0.04

In general, helium tends to produce the lowest LODs (typically <1 ng) compared with using nitrogen or argon as the ionisation gas. Other publications using nitrogen as the ionisation gas have found it to have LODs an order of magnitude worse than those produced by helium, which is the general trend seen in this work.^8^, ^35^ Sisco et al used nitrogen as the ionisation gas with a thermal desorption DART set‐up and reported estimated LODs for 34 drugs, metabolites and cutting agents using a JEOL JMS‐T100LP AccuTOF MS. The work reported in this study is in close agreement with the approximate sensitivity published for amphetamine (1.0 ng), cocaine (0.25 ng) and THC (1.0 ng).^33^ The similarity in LODs is surprising considering the difference in performance and resolution of the instruments used (time‐of‐flight vs. single quadrupole). These data, presented in Table 3, suggest that nitrogen may not be suitable for use if detection limits need to be relatively low (<10 ng), particularly for amino acids. As mentioned earlier, these values are based on the blank response, and argon produced a much lower response, whereas nitrogen tended to produce the highest values, so it fits that the blank response would also be greater having a knock‐on effect on the perceived LOD. The calculation used to establish LODs is also likely responsible for the low LOD calculated for ketamine with argon gas, due to a very low blank response observed for the ion at *m/z* 238 used to quantify ketamine. Research has shown that using a smaller aperture (0.5 mm) cap can reduce the presence of NO^+^ seen in positive N_2_‐DART spectra, resulting in less oxidation products being formed.^36^ This could reduce the background signal observed and lead to increased production of protonated molecules which may improve the LODs. Dopants such as dichloromethane or ammonium nitrate which produce anions Cl^−^ and NO_3_^−^ could improve the detection limits of the explosives PETN, RDX and tetryl as adducts may be encouraged to form. Anions NO_3_^−^ and NO_2_^−^ can form from the DART gas stream, as demonstrated in this work; however, their production is limited by the analyte concentration.

## 4 CONCLUSION

This study has demonstrated that repeatable analyses of drugs, explosives and amino acids can be achieved using DART‐MS. Although limited by sensitivity (when compared to He‐DART‐MS) using high purity N_2_ as the ionisation gas can provide a robust analysis with nanogram sensitivity and acceptable linearity showing potential utility for semi‐quantitative analysis. The LODs on this single quadrupole system compare favourably with those observed elsewhere using more complex and sensitive instrumentation.^33^

Due to the available energetic species, Ar‐DART needs to use an intermediate organic vapour to provide a viable analysis; however, using ethanol can generate ions similar to He‐DART and in contrast to N_2_‐DART where additional oxidation products are formed.

All the analytes could be ionised and detected using helium, nitrogen or argon as the ionisation gas for DART. Both helium and nitrogen were more efficient at ionising analytes compared to argon gas; however, improvements could be made by changing the dopant solvent and by having a constant flow via a syringe pump. Yang et al explored a variety of different dopant solvents and found success with methanol, fluorobenzene, ethanol and acetone.^18^ Although ethanol is a suitable dopant choice for proton transfer DART, it is weakly ionised and thus results in lower detection limits than He and N_2_ DART. Other dopants are more effectively ionised but have different proton affinities and therefore different selectivity.

Helium generally provided higher levels of sensitivity compared with nitrogen and argon; however, nitrogen produced the largest response to concentration. Nitrogen can therefore be used as a greener alternative to helium for analysis by DART, providing reasonable limits of detection, good linearity and reproducibility even without the use of an internal standard.

### PEER REVIEW

The peer review history for this article is available at https://www.webofscience.com/api/gateway/wos/peer-review/10.1002/rcm.9521.

## Supporting information


**TABLE S1** SIM method voltages used for each ionisation gas for all analytes tested with assigned *m/z* value and associated species. The most abundant ion is highlighted with an * for each analyte
**FIGURE S1** Photograph of DART® source coupled to the Waters QDa mass spectrometer
**FIGURE S2** Photograph of OpenSpot® Card
**FIGURE S3** Negative ion mode background spectra for A, He (helium), B, N_2_ (nitrogen) and C, Ar (argon) ionisation gases with background ions for oxygen, nitrate and nitrite ions labelled
**FIGURE S4** Positive ion mode background spectra for A, He (helium), B, N_2_ (nitrogen) and C, Ar (argon) ionisation gases
**FIGURE S5** Example of positive ionisation spectra for Amphetamine with A, He (helium), B, N_2_ (nitrogen), C, Ar (argon) ionisation gases with *m/*z values identified and species assigned
**FIGURE S6** Calibration curve (n = 5) for amphetamine produced with argon as ionisation gas. Red square is an outlier and not included in the line of best fit
**FIGURE S7** Calibration curve (n = 5) for cocaine produced with argon as ionisation gas. Red square is an outlier and not included in the line of best fit
**FIGURE S8** Calibration curve (n = 5) for ketamine produced with argon as ionisation gas. Red square is an outlier and not included in the line of best fit
**FIGURE S9** Calibration curve (n = 5) for THC produced with argon as ionisation gas. Red square is an outlier and not included in the line of best fit
**FIGURE S10** Calibration curve (n = 5) for TNT produced with argon as ionisation gas. Red square is an outlier and not included in the line of best fit
**FIGURE S11** Calibration curve (n = 5) for RDX produced with argon as ionisation gas. Red square is an outlier and not included in the line of best fit
**TABLE S2** RSDs (%) where n = 5 for all analytes across all calibrator levels used for calibration curves for each ionisation gas (calculated as shown on page 10). An “‐“indicates a value could not be calculated for this concentration
**TABLE S3** Back‐calculated accuracy using the equation of the line for all calibrator points at all concentrations (as discussed on page 10). Legend: Red indicates a negative concentration was produced. Blue indicates a point which was not included in the calibration line due to it being a statistical outlier. Purple indicates points which did not fit within the linear dynamic range. An “‐” indicates a value could not be calculated for this concentration.

## Data Availability

The data that supports the findings of this study is openly available in Open Science Framework at DOI: 10.17605/OSF.IO/PB3AF.
