# Supplementary material for: Direct analysis in real‐time mass spectrometry: Observations of helium, nitrogen and argon as ionisation gas for the detection of small molecules using a single quadrupole instrument
Source: Rapid Commun Mass Spectrom. 2023 May 3;37(12):e9521. doi: 10.1002/rcm.9521 (PMC10909476; doi:10.1002/rcm.9521)
Supplement: Supplementary file 1 — TABLE S1 SIM method voltages used for each ionisation gas for all analytes tested with assigned m/z value and associated species. The most abundant ion is highlighted with an * for each analyte FIGURE S1 Photograph of DART® source coupled to the Waters QDa mass spectrometer FIGURE S2 Photograph of OpenSpot® Card FIGURE S3 Negative ion mode background spectra for A, He (helium), B, N2 (nitrogen) and C, Ar (argon) ionisation gases with background ions for oxygen, nitrate and nitrite ions labelled FIGURE S4 Positive ion mode background spectra for A, He (helium), B, N2 (nitrogen) and C, Ar (argon) ionisation gases FIGURE S5 Example of positive ionisation spectra for Amphetamine with A, He (helium), B, N2 (nitrogen), C, Ar (argon) ionisation gases with m/z values identified and species assigned FIGURE S6 Calibration curve (n = 5) for amphetamine produced with argon as ionisation gas. Red square is an outlier and not included in the line of best fit FIGURE S7 Calibration curve (n = 5) for cocaine produced with argon as ionisation gas. Red square is an outlier and not included in the line of best fit FIGURE S8 Calibration curve (n = 5) for ketamine produced with argon as ionisation gas. Red square is an outlier and not included in the line of best fit FIGURE S9 Calibration curve (n = 5) for THC produced with argon as ionisation gas. Red square is an outlier and not included in the line of best fit FIGURE S10 Calibration curve (n = 5) for TNT produced with argon as ionisation gas. Red square is an outlier and not included in the line of best fit FIGURE S11 Calibration curve (n = 5) for RDX produced with argon as ionisation gas. Red square is an outlier and not included in the line of best fit TABLE S2 RSDs (%) where n = 5 for all analytes across all calibrator levels used for calibration curves for each ionisation gas (calculated as shown on page 10). An “‐“indicates a value could not be calculated for this concentration TABLE S3 Back‐calculated accuracy using the equation [file RCM-37-e9521-s001.docx]

**Supporting information for**

**Direct analysis in real time mass spectrometry: Observations of helium, nitrogen and argon as ionisation gas**

Simone Mathias and Patrick Sears^*^

*Corresponding author: [p.sears@surrey.ac.uk](mailto:p.sears@surrey.ac.uk)

School of Chemistry and Chemical Engineering, University of Surrey, Guildford, UK

**Contents**

Page 2: Table S1: SIM method voltages used for each ionisation gas for all analytes tested with assigned m/z value and associated species. The most abundant ion is highlighted with an * for each analyte.

Page 3: Figure S1: Photograph of DART® source coupled to the Waters QDa mass spectrometer.

Page 3: Figure S2: Photograph of OpenSpot® Card

Page 4: Figure S3: Negative ion mode background spectra for A He (helium), B N_2_ (nitrogen) and C Ar (argon) ionisation gases with background ions for oxygen, nitrate and nitrite ions labelled.

Page 5: Figure S4: Positive ion mode background spectra for A He (helium), B N_2_ (nitrogen) and C Ar (argon) ionisation gases.

Page 6: Figure S5: Example of positive ionisation spectra for Amphetamine with A He (helium), B N_2_ (nitrogen), C Ar (argon) ionisation gases with m/z values identified and species assigned.

Page 7: Figure S6: Calibration curve (n=5) for amphetamine produced with argon as ionisation gas.

Page 7: Figure S7: Calibration curve (n=5) for cocaine produced with argon as ionisation gas.

Page 8: Figure S8: Calibration curve (n=5) for ketamine produced with argon as ionisation gas.

Page 8: Figure S9: Calibration curve (n=5) for THC produced with argon as ionisation gas

Page 9: Figure S10: Calibration curve (n=5) for TNT produced with argon as ionisation gas.

Page 9: Figure S11: Calibration curve (n=5) for RDX produced with argon as ionisation gas.

Page 10: Equations and calculations used within the manuscript

Page 11: Table S2: RSDs (%) where n=5 for all analytes across all calibrator levels used for calibration curves for each ionisation gas (calculated as shown on page 10). An “-“ indicates a value could not be calculated for this concentration.

Page 12: Table S3: Back calculated accuracy using the equation of the line for all calibrator points at all concentrations (as discussed on page 10). Legend: Red indicates a negative concentration was produced. Blue indicates a point which was not included in the calibration line due to it being a statistical outlier. Purple indicates points which did not fit within the linear dynamic range. An “-“ indicates a value could not be calculated for this concentration.

**Table S1:** SIM method voltages used for each ionisation gas for all analytes tested with assigned *m/z* value and associated species. The most abundant ion is highlighted with an * for each analyte.

| **Analyte** | ***m/z*** | **Assignment** | **Helium Cone Voltage (V)** | **Nitrogen Cone Voltage (V)** | **Argon Cone Voltage** |
| --- | --- | --- | --- | --- | --- |
| **Amphetamine** | 136* | [M+H]^+^ | 25 | 20 | 15 |
|  | 119 | [M-NH_2_]^+^ | 50 | 20 | 15 |
|  | 91 | [M-NH_2_-C_2_H_3_]^+^ | 80 | 50 | 20 |
| **Ketamine** | 240 | [M(^37^Cl)+H]^+^ | 20 | 15 | 15 |
|  | 238* | [M(^35^Cl)+H]^+^ | 20 | 15 | 15 |
|  | 220 | [M-HO]^+^ | 55 | 30 | 25 |
|  | 207 | [M-CNH_4_]^+^ | 50 | 30 | 30 |
|  | 179 | [M-CNH_4_-CO]^+^ | 55 | 35 | 30 |
|  | 125 | [M-CNH_4_-CO-C_4_H_6_]^+^ | 75 | 55 | 40 |
| **THC** | 315* | [M+H]^+^ | 20 | 5 | 10 |
|  | 259 | [M-C_4_H_7_]^+^ | 60 | 45 | 40 |
|  | 235 | [M-C_6_H_7_]^+^ | 70 | 35 | 40 |
|  | 193 | [M-C_9_H_13_]^+^ | 45 | 50 | 40 |
| **Cocaine** | 304* | [M+H]^+^ | 10 | 10 | 10 |
|  | 182 | [M-C_7_H_5_O_2_]^+^ | 35 | 40 | 45 |
|  | 105 | [M-C_10_H_16_NO_3_]^+^ | 55 | 55 | 60 |
|  | 82 | [M-C_12_H_13_O_4_]^+^ | 55 | 55 | 55 |
|  | 77 | [M-C_11_H_16_NO_4_]^+^ | 70 | 80 | 75 |
| **Phenylalanine** | 166* | [M+H]^+^ | 35 | 20 | 20 |
|  | 120 | [M-CHO_2_]^+^ | 70 | 40 | 20 |
|  | 103 | [M-CH_4_NO_2_]^+^ | 80 | 55 | 40 |
| **Leucine** | 132* | [M+H]^+^ | 20 | 15 | 20 |
|  | 86 | [M-CH­_2_O_2_]^+^ | 60 | 35 | 40 |
| **HMTD** | 224 | [TMDDD+NH_4_]^+^ | 10 | 10 | 10 |
|  | 209*(He & N_2_) | [M+H]^+^ | 5 | 5 | 5 |
|  | 207*(Ar) | [TMDDD+H]^+^ | 20 | 20 | 20 |
|  | 179 | [TMDDD-CO]^+^ | 5 | 5 | 5 |
|  | 145 | [TMDDD-H_2_O_2_]^+ ­^or [M-CH_4_O_3_]^+^ | 10 | 10 | 10 |
|  | 88 | [TMDDD-C_3_H_5_NO_4_]^+^ or [M-C_3_H_7_NO_4_]^+^ | 20 | 20 | 20 |
| **TNT** | 227 | [M•]^-^ | 10 | 5 | 15 |
|  | 226* | [M-H]^-^ | 5 | 5 | 15 |
|  | 213 | [M+O-NO]^-^ | 15 | 10 | 15 |
|  | 210 | [M-HO]^-^ | 20 | - | 20 |
|  | 197 | [M-NO]^-^ | 20 | 5 | 5 |
| **RDX** | 324 | [M+C_2_H_4_N_3_O_2_]^-^ | 10 | - | - |
|  | 284 | [M+NO_3_]^-^ | 5 | 5 | 5 |
|  | 268* | [M+NO_2_]^-^ | 5 | 5 | 5 |
|  | 267 | [M+NO_2_-H]^-^ | 5 | 5 | 5 |
|  | 129 | [M-HN_2_O_4_]^-^ | 20 | - | - |
|  | 102 | [M-CH­_2_N_3_O_4_]^-^ | 15 | - | - |
| **Tetryl** | 349 | [M+NO_3_]^-^ | 5 | 5 | - |
|  | 304 | [M-NO_2_+HNO_3_]^-^ | 15 | 10 | 5 |
|  | 288 | [M-NO_2_+HNO_2_]^-^ | - | - | 5 |
|  | 257 | [M-NO]^-^ | 5 | 5 | 5 |
|  | 241* | [M-NO_2_]^-^ | 5 | 5 | 5 |
|  | 181 | [M-NO_2_-CH_2_ NO_2_]^-^ | 20 | 20 | 15 |
| **PETN** | 378* | [M+NO_3_]^-^ | 5 | 5 | 5 |
|  | 362 | [M+NO_2_]^-^ | 5 | - | 5 |
|  | 315 | [M-H]^-^ | 5 | 5 | 5 |
|  | 62 | [NO­_3_]^-^ | 10 | - | 20 |


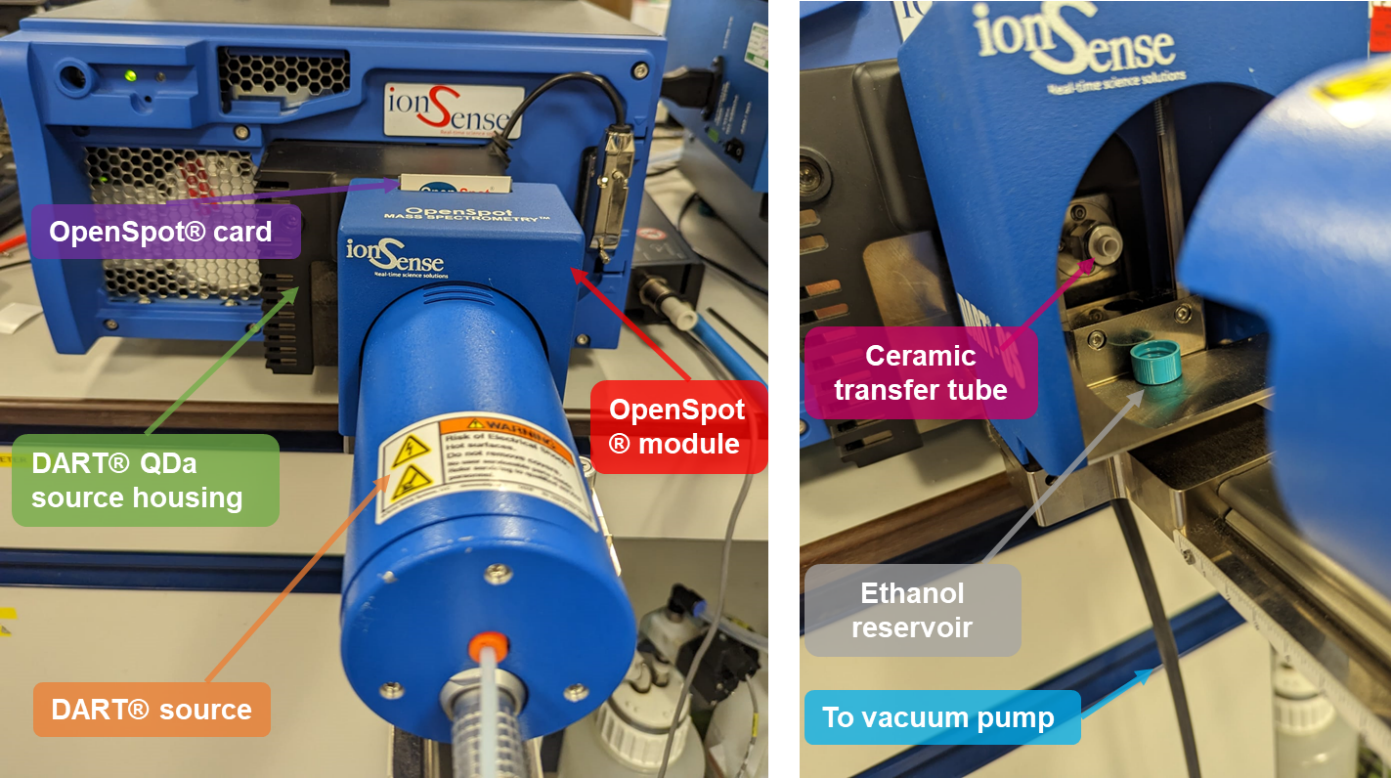


**Figure S1:** Photograph of DART® source coupled to the Waters QDa mass spectrometer.


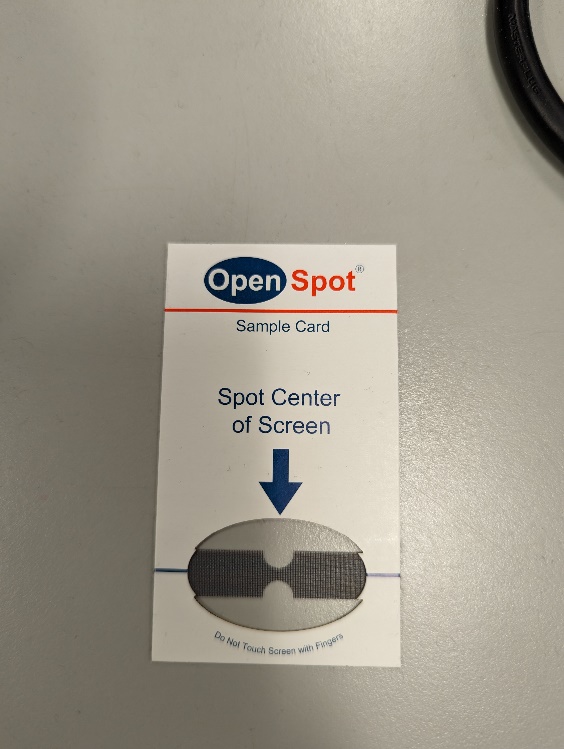


**Figure S2:** Photograph of OpenSpot® Card.


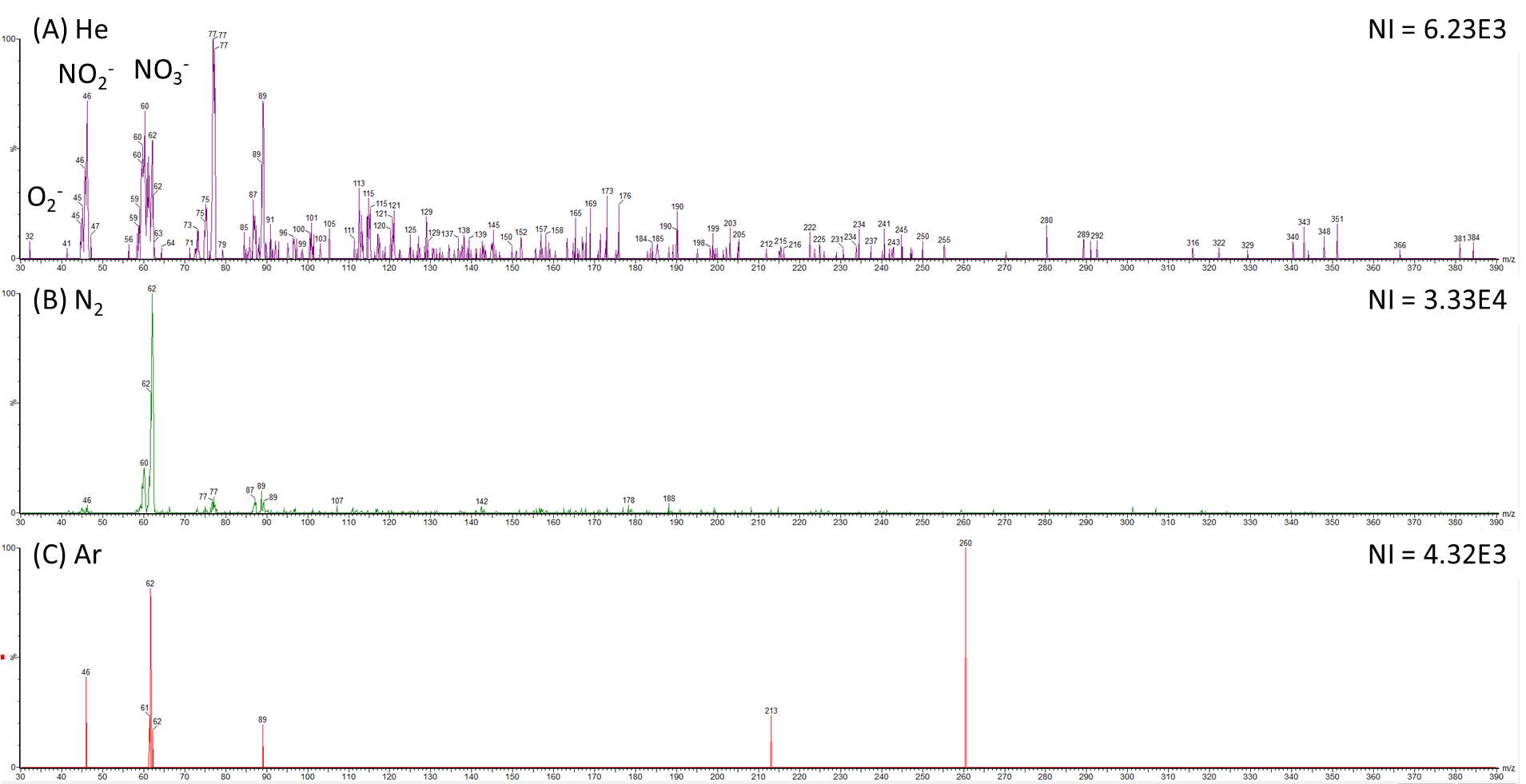


**Figure S3:** Negative ion mode background spectra for A He (helium), B N_2_ (nitrogen) and C Ar (argon) ionisation gases with background ions for oxygen, nitrate and nitrite ions labelled.


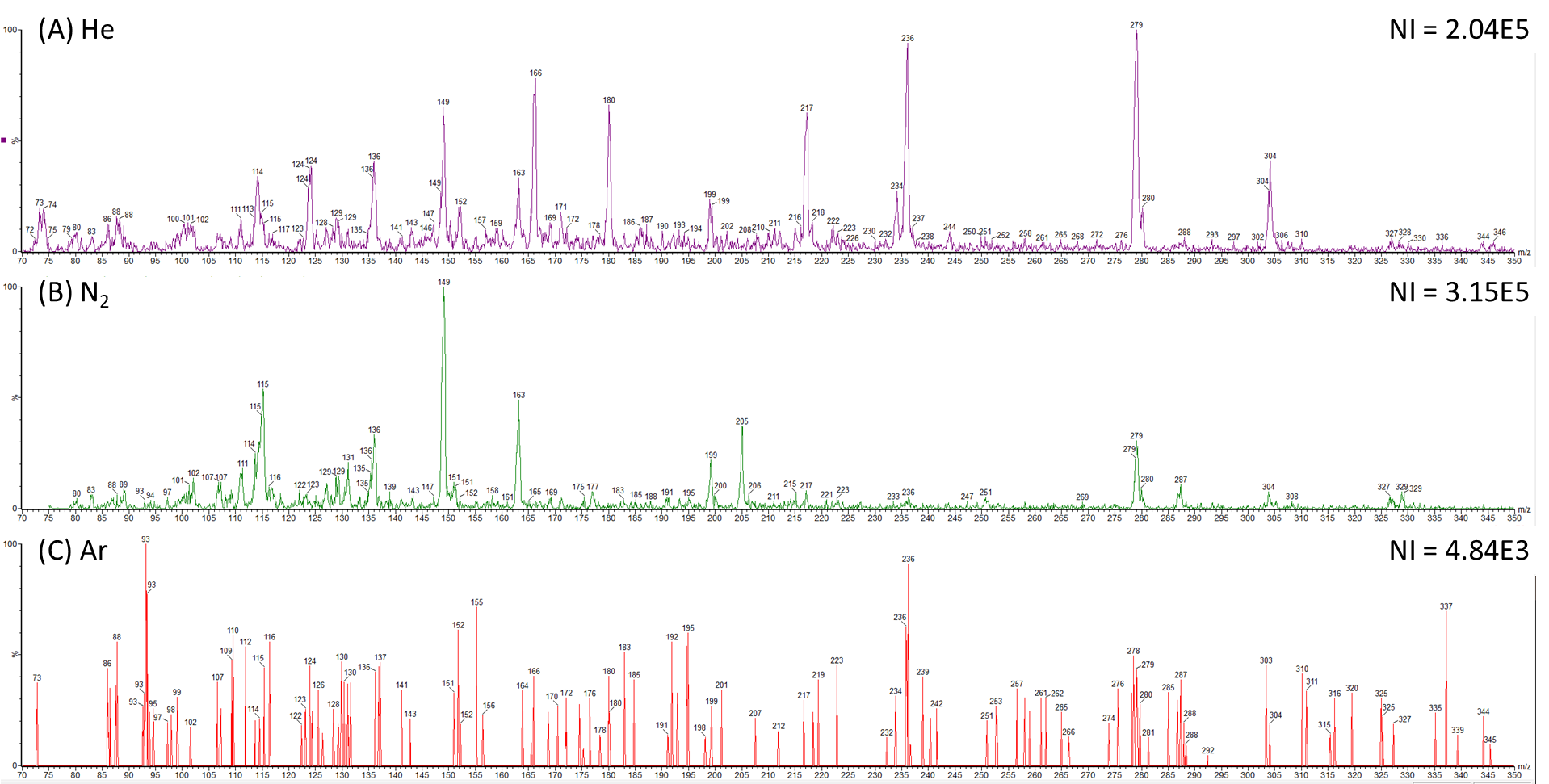


**Figure S4:** Positive ion mode background spectra for A He (helium), B N_2_ (nitrogen) and C Ar (argon) ionisation gases.

**
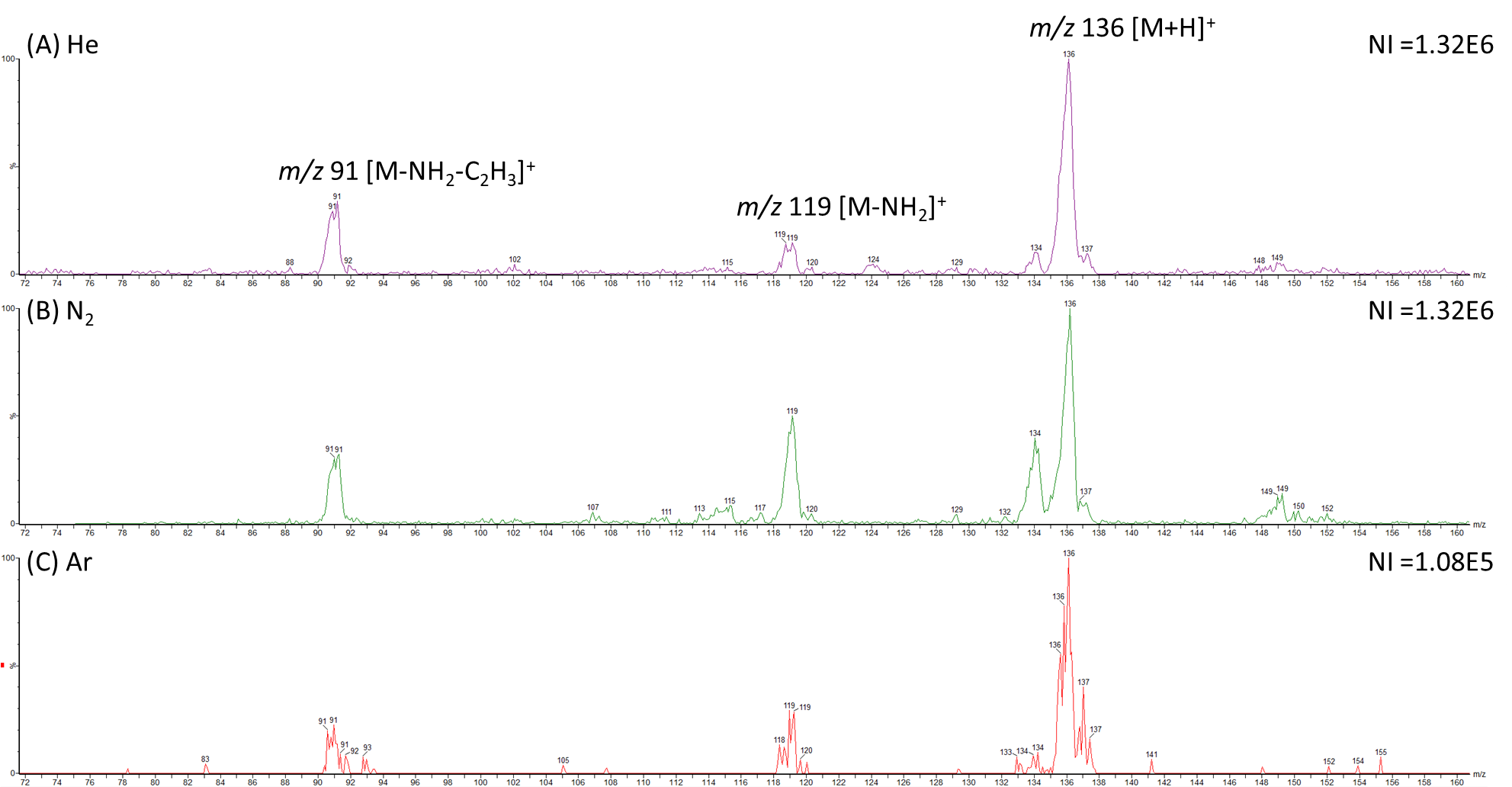
**

**Figure S5:** Example of positive ionisation spectra for Amphetamine with A He (helium, B N_2_ (nitrogen), C Ar (argon) ionisation gases with *m/*z values identified and species assigned.

**Figure S6:** Calibration curve (n=5) for amphetamine produced with argon as ionisation gas. Red square is an outlier and not included in the line of best fit.

**Figure S7:** Calibration curve (n=5) for cocaine produced with argon as ionisation gas. Red square is an outlier and not included in the line of best fit.

**Figure S8:** Calibration curve (n=5) for ketamine produced with argon as ionisation gas. Red square is an outlier and not included in the line of best fit.

**Figure S9:** Calibration curve (n=5) for THC produced with argon as ionisation gas. Red square is an outlier and not included in the line of best fit.

**Figure S10:** Calibration curve (n=5) for TNT produced with argon as ionisation gas. Red square is an outlier and not included in the line of best fit.

**Figure S11:** Calibration curve (n=5) for RDX produced with argon as ionisation gas. Red square is an outlier and not included in the line of best fit.

**Equations & Calculations**

**Calculating outliers on a line of best fit –**

Calculate the predicted y value ($\hat{y_{i}}$) using the following calculation = $\hat{y_{i}}=m* X+C$ using the proposed equation of the line with the suspected outlier removed.

Where m is the slope, X is the independent variable (in this case concentration) and C is the intercept.

The predicted value is then used to calculate the residual values ($r_{i}$) using the following calculation $r_{i}=y- \hat{y_{i}}$ where y is the actual Y value and $\hat{y_{i}}$ is the predicted y value.

The sum of the squared errors (SSE) is then calculated by squaring each residual value = ${r_{i}}^{2}$ and adding them together.

The standard deviation of the residual can then be calculated from the SSE using the following equation:

$$s= \sqrt{\frac{SSE}{n-2}}$$

Where s is the standard deviation and n is equal to the total number of data points.

If an actual Y value was greater or smaller than ± 2(s), was considered to be an outlier and removed from the line of best fit.

**Calculating the LOD**

$$LOD= X_{b1}+{3S}_{b1}$$

Where X_b1_ is the mean blank response and S_b1_ is the standard deviation of the blank. Analyte is considered detected when the response is above the value given by the above equation.

**Back-calculated accuracy**

The equation of the regression line (y = mx + C) was rearranged to calculate the calculated concentration for each average peak area corresponding to a target concentration for all analytes across all ionisation gases. This calculated concentration was then divided by the target concentration and multiplied by 100 to give a percentage accuracy.

**Relative Standard Deviation**

$$RSD \%= \frac{S}{\bar{x}} \times100$$

Where S is the standard deviation and $\bar{x}$ is the mean.

**Table S2:** RSDs (%) where n=5 for all analytes across all calibrator levels used for calibration curves for each ionisation gas (calculated as shown on page 10). An “-“ indicates a value could not be calculated for this concentration.

| **Analyte** | **Ionisation gas** | **Concentration (ng)** | | | | | | | |
| --- | --- | --- | --- | --- | --- | --- | --- | --- | --- |
|  |  | **5** | **10** | **15** | **20** | **25** | **50** | **75** | **100** |
| **Amphetamine** | **He** | 9% | 13% | 23% | 17% | 17% | 35% | 31% | 32% |
|  | **N_2_** | 12% | 19% | 20% | 13% | 16% | 32% | 22% | 17% |
|  | **Ar** | 14% | 57% | 41% | 24% | 12% | 15% | 19% | 37% |
| **Ketamine** | **He** | 25% | 26% | 12% | 20% | 26% | 27% | 21% | 19% |
|  | **N_2_** | 16% | 15% | 14% | 17% | 22% | 23% | 14% | 23% |
|  | **Ar** | 59% | 62% | 42% | 56% | 47% | 43% | 58% | 21% |
| **Cocaine** | **He** | 31% | 25% | 20% | 34% | 22% | 18% | 15% | 25% |
|  | **N_2_** | 10% | 25% | 3% | 24% | 6% | 26% | 26% | 20% |
|  | **Ar** | 50% | 39% | 27% | 44% | 38% | 48% | 48% | 52% |
| **THC** | **He** | 32% | 30% | 25% | 27% | 20% | 34% | 18% | 13% |
|  | **N_2_** | 63% | 40% | 41% | 77% | 60% | 12% | 64% | 70% |
|  | **Ar** | 75% | 13% | 23% | 51% | 36% | 29% | 31% | 40% |
| **Phenylalanine** | **He** | 37% | 49% | 27% | 18% | 5% | 16% | 14% | 16% |
|  | **N_2_** | - | 52% | 53% | 27% | 29% | 19% | 22% | 26% |
|  | **Ar** | 105% | 54% | 29% | 39% | 17% | 35% | 27% | 27% |
| **Leucine** | **He** | 50% | 23% | 28% | 15% | 5% | 11% | 12% | 9% |
|  | **N_2_** | - | 14% | 10% | 23% | 30% | 18% | 17% | 6% |
|  | **Ar** | 41% | 58% | 28% | 28% | 21% | 25% | 24% | 13% |
| **HMTD** | **He** | 29% | 16% | 41% | 13% | 16% | 13% | 19% | 9% |
|  | **N_2_** | 29% | 18% | 16% | 25% | 16% | 9% | 17% | 12% |
|  | **Ar** | - | 48% | 18% | 24% | 11% | 32% | 70% | - |
| **TNT** | **He** | 21% | 18% | 9% | 36% | 2% | 20% | 10% | 13% |
|  | **N_2_** | 16% | 18% | 22% | 9% | 23% | 28% | 11% | 27% |
|  | **Ar** | 45% | 55% | 26% | 35% | 78% | 51% | 51% | 43% |
| **RDX** | **He** | 44% | 35% | 41% | 7% | 36% | 23% | 22% | 20% |
|  | **N_2_** | 16% | 7% | 6% | 28% | 27% | 17% | 17% | 37% |
|  | **Ar** | 13% | 21% | 28% | 39% | 34% | 35% | 40% | 29% |
| **Tetryl** | **He** | 30% | 32% | 18% | 23% | 32% | 15% | 25% | 29% |
|  | **N_2_** | 12% | 17% | 32% | 19% | 22% | 13% | 25% | 11% |
|  | **Ar** | 40% | 19% | 31% | 11% | 24% | 19% | 27% | 31% |
| **PETN** | **He** | 40% | 94% | 64% | 27% | 45% | 28% | 24% | 31% |
|  | **N_2_** | 22% | 12% | 3% | 15% | 11% | 5% | 10% | 14% |
|  | **Ar** | 32% | 29% | 30% | 18% | 36% | 36% | 45% | 21% |

**Table S3:** Back calculated accuracy using the equation of the line for all calibrator points at all concentrations (as discussed on page 10). Legend: Red indicates a negative concentration was produced. Blue indicates a point which was not included in the calibration line due to it being a statistical outlier. Purple indicates points which did not fit within the linear dynamic range. An “-“ indicates a value could not be calculated for this concentration.

| **Analyte** | **Ionisation gas** | **Concentration (ng)** | | | | | | | |
| --- | --- | --- | --- | --- | --- | --- | --- | --- | --- |
|  |  | **5** | **10** | **15** | **20** | **25** | **50** | **75** | **100** |
| **Amphetamine** | **He** | 50% | 144% | 127% | 51% | 93% | 143% | 91% | 96% |
|  | **N_2_** | 150% | 64% | 144% | 116% | 123% | 120% | 87% | 100% |
|  | **Ar** | 4% | 135% | 98% | 97% | 415% | 122% | 82% | 105% |
| **Ketamine** | **He** | 60% | 56% | 161% | 127% | 115% | 98% | 85% | 107% |
|  | **N_2_** | 100% | 45% | 119% | 110% | 108% | 121% | 120% | 83% |
|  | **Ar** | 96% | 178% | 200% | 78% | 57% | 68% | 53% | 109% |
| **Cocaine** | **He** | 85% | 10% | 159% | 134% | 137% | 88% | 82% | 100% |
|  | **N_2_** | 32% | 52% | 103% | 111% | 133% | 113% | 95% | 98% |
|  | **Ar** | 14% | 62% | 121% | 59% | 160% | 223% | 101% | 97% |
| **THC** | **He** | 166% | 152% | 105% | 134% | 112% | 107% | 88% | 103% |
|  | **N_2_** | 101% | 80% | 105% | 112% | 93% | 103% | 99% | 52% |
|  | **Ar** | 67% | 82% | 257% | 53% | 130% | 110% | 114% | 90% |
| **Phenylalanine** | **He** | 58% | 48% | 94% | 108% | 125% | 110% | 94% | 100% |
|  | **N_2_** | - | 98% | 106% | 109% | 92% | 103% | 94% | 103% |
|  | **Ar** | 85% | 93% | 98% | 115% | 101% | 87% | 112% | 96% |
| **Leucine** | **He** | 42% | 86% | 110% | 97% | 113% | 92% | 115% | 93% |
|  | **N_2_** | 45% | 52% | 84% | 76% | 119% | 114% | 116% | 88% |
|  | **Ar** | 5% | 74% | 93% | 78% | 132% | 117% | 108% | 91% |
| **HMTD** | **He** | 131% | 119% | 83% | 99% | 130% | 80% | 92% | 108% |
|  | **N_2_** | 67% | 106% | 94% | 98% | 116% | 96% | 100% | 100% |
|  | **Ar** | - | 75% | 42% | 214% | 91% | 63% | 112% | - |
| **TNT** | **He** | 48% | 67% | 94% | 76% | 132% | 112% | 105% | 94% |
|  | **N_2_** | 5% | 91% | 94% | 111% | 115% | 111% | 93% | 70% |
|  | **Ar** | 99% | 89% | 377% | 198% | 28% | 135% | 114% | 91% |
| **RDX** | **He** | 21% | 91% | 155% | 82% | 108% | 106% | 86% | 106% |
|  | **N_2_** | 9% | 95% | 93% | 96% | 96% | 113% | 109% | 97% |
|  | **Ar** | 151% | 134% | 87% | 56% | 228% | 110% | 105% | 97% |
| **Tetryl** | **He** | 76% | 13% | 160% | 131% | 132% | 92% | 98% | 67% |
|  | **N_2_** | 85% | 13% | 99% | 124% | 126% | 130% | 103% | 90% |
|  | **Ar** | 100% | 81% | 123% | 94% | 98% | 44% | 65% | 47% |
| **PETN** | **He** | 629% | 7% | 108% | 100% | 169% | 72% | 99% | 103% |
|  | **N_2_** | 27% | 97% | 99% | 104% | 115% | 112% | 99% | 97% |
|  | **Ar** | 581% | 239% | 270% | 103% | 68% | 114% | 110% | 92% |
